# Trainee led research networks in the implementation and delivery of multicentre randomised trials: a model from the west midlands research collaborative

**DOI:** 10.1186/1745-6215-14-S1-O110

**Published:** 2013-11-29

**Authors:** Aneel Bhangu, Thomas Pinkney, Laura Magill, Dion Morton

**Affiliations:** 1 West Midlands Research Collaborative, Birmingham, UK

## Background

The timely delivery of randomised controlled trials across multicentre settings remains challenging. Trainees form a natural rotating network, covering most hospitals in wide geographical regions. We report the benefits of such a network with the subsequent development of a national trainee-led model.

## Methods

The West Midlands Research Collaborative is a trainee-led research group. Based around rotating general surgery registrars, the group has devised, planned and delivered multicentre randomised trials. This structure has led to the development of other regional groups, which has now enabled national geographical coverage. The trainee-led approach has been mimicked in other surgical disciplines, and is emerging in some medical specialties.

## Results

Trainees from the West Midlands Research Collaborative have completed a randomised trial of over 750 patients, is close to completing a second trial of 950 patients and has recently commenced a third trial aiming for 560 patients. Development of the national structure has delivered a multicentre audit of 95 hospitals, which was devised and completed within six months. Novel strategies that have led to these successes include widespread targeting of relevant health care professionals (e.g. coffee room guerrilla tactics to engage consultants) and inclusion of patients from previously difficult to access sources (e.g. pre-operative assessment clinics and emergency settings).

## Conclusion

This trainee-led collaborative research model has been proved feasible and has delivered high quality randomised controlled trials. The structure shown could now be applied at a national and international level, and across all medical and surgical specialties.

